# Blood lead concentrations and attention deficit hyperactivity disorder in Korean children: a hospital-based case control study

**DOI:** 10.1186/s12887-016-0696-5

**Published:** 2016-09-22

**Authors:** Jae Hong Park, Ju-Hee Seo, Young-Seoub Hong, Yu-Mi Kim, Je-Wook Kang, Jae-Ho Yoo, Hee Won Chueh, Jung Hyun Lee, Min Jung Kwak, Jeongseon Kim, Hae Dong Woo, Dong Woo Kim, Young Rong Bang, Byeong Moo Choe

**Affiliations:** 1 Department of Psychiatry, College of Medicine, Dong-A University, Dong-A University Hospital, 26 Daesingongwon-ro, Seo-gu, Busan 602-715 Republic of Korea; 2 Heavy Metal Exposure Environmental Health Center, Dong-A University, 32, Daesingongwon-ro, Seo-gu, Busan 602-714 Republic of Korea; 3 Department of Preventive Medicine, College of Medicine, Dong-A University, Dong-A University Hospital, 26, Daesingongwon-ro, Seo-gu, Busan 602-715 Republic of Korea; 4 Department of Child and Adolescent Psychiatry, College of Medicine, Inje University Busan Paik Hospital, 75 Bokji-ro, Busanjin-gu, Busan 614-735 Republic of Korea; 5 Department of Pediatrics, College of Medicine, Dong-A University, Dong-A University Hospital, 26 Daesingongwon-ro, Seo-gu, Busan 602-715 Republic of Korea; 6 Department of Pediatrics, Kosin University Gospel Hospital, 262, Gamcheon-ro, Seo-gu, Busan 602-702 Republic of Korea; 7 Department of Pediatrics, Pusan National University Hospital, Pusan National University School of Medicine, 179, Gudeok-ro, Seo-gu, Busan 602-739 Republic of Korea; 8 Molecular Epidemiology Branch, National Cancer Center, 323 Ilsan-ro, Ilsandong-gu, Goyang-si, Gyeonggi-do 410-769 Republic of Korea; 9 Department of Home Economics, College of Natural Science, Korea National Open University, 86, Daehak-ro, Jongno-gu, Seoul 110-791 Republic of Korea

**Keywords:** ADHD, Child, Environment, Heavy Metal, Lead

## Abstract

**Background:**

Because the developing brain of a child is vulnerable to environmental toxins, even very low concentration of neurotoxin can affect children’s neurodevelopment. Lead is a neurotoxic heavy metal which has the harmful effect on the striatal-frontal circuit of brain. This area of the brain is known to be closely related to attention deficit hyperactivity disorder (ADHD) pathophysiology. The primary objective of the present study was to investigate whether elevated blood lead concentration is a risk factor for ADHD. The secondary objective was to examine the association between blood lead concentration and symptom severity.

**Methods:**

We conducted a frequency-matched, hospital-based case-control study with 114 medically diagnosed ADHD cases and 114 controls. The participants were matched for age and sex. The diagnoses of ADHD were assessed with semi-structured diagnostic interviews. The participants completed the continuous performance test (CPT), and their parents completed the ADHD-rating scale (ADHD-RS). Blood lead concentrations were measured by using graphite furnace atomic absorption spectrometry featuring Zeeman background correction.

**Results:**

Children with ADHD exhibited blood lead concentrations that were significantly higher than those of the controls ( 1.90 ± 086 μg/dℓ vs. 1.59 ± 0.68 μg/dℓ, *p* = 0.003). The log transformed total blood lead concentration was associated with a higher risk of ADHD (OR: 1.60, 95 % CI: 1.04–2.45, *p* < 0.05). The analysis also revealed that the children with blood lead concentrations above 2.30 μg/dℓ were at a 2.5–fold (95 % CI: 1.09–5.87, *p* < 0.05) greater risk of having ADHD. After adjusting for covariates, our multivariate regression models indicated that blood lead concentrations were not significantly associated with ADHD-RS or CPT profiles among the ADHD cases.

**Conclusion:**

Even low blood lead concentrations are a risk factor for ADHD in children. This study warrants primary prevention policies to reduce the environmental lead burden. Future studies may be required to ascertain the effects of lead on symptom severity in ADHD.

## Background

Attention deficit hyperactivity disorder (ADHD) is a neurodevelopmental disorder characterized by persistent and developmentally inappropriate expressions of inattention, impulsivity, and hyperactivity. ADHD is the most common neurodevelopmental disorder in childhood, with a worldwide prevalence of approximately 5 % [1, 2]. Both genetic and environmental factors are involved in the etiology of ADHD. There is robust evidence for the strong inheritance of ADHD, with a genetic heritability of approximately 75 % [3]. However, the environmental risk factors for ADHD, which constitute 25 % of the etiology of ADHD, remain unclear.

Among the environmental factors that are suspected to be associated with ADHD, lead exposure has been implicated in the etiology of ADHD [4]. Lead is a neurotoxic heavy metal that is widely present in the environment. Common sources of lead exposure include lead-based paint from buildings and toys, vehicle exhaust fumes, water from leaded pipes, secondhand smoke, and air pollution. Because the nervous systems of children are more vulnerable than those of adults to the neurotoxic effects of lead, even low levels of lead can affect neurodevelopment in children [5]. Children absorb lead more readily than adults, and lead crosses the blood-brain barrier more easily in children [6]. Some studies documented a link between lead and ADHD pathophysiology. The brain regions that are most vulnerable to lead exposure are the prefrontal cortex, basal ganglia, hippocampus, and cerebellum [6]. Dysfunctions of these regions have been postulated to be involved in ADHD pathophysiology [7]. Findings from animal studies have demonstrated that lead exposure affect dopamine metabolism and decrease dopamine receptor binding in the striatum [8, 9]. Reduced dopamine activity in striatum has been implicated in the core symptoms of ADHD [10, 11]. Overall, these studies revealed that lead adversely affects the dopamine system in the prefrontal-striatal network, which is linked to the core pathophysiology of ADHD.

Previous studies have reported that even low levels (<5 μg/dℓ) of lead exposure are associated with inattention and hyperactivity/impulsivity, which are the two core symptom domains of ADHD [12–15]. However, many of the previous studies focused on ADHD-like symptoms (i.e., inattention and hyperactivity/impulsivity) rather than categorical diagnoses of ADHD. These previous studies analyzed symptom-level measures of inattention and hyperactivity/impulsivity. Case-control studies that investigated whether lead is associated with medically diagnosed ADHD are still limited.

Another issue is whether blood lead concentrations are associated with symptom severity in ADHD. Previous population-based studies have reported inconsistent results regarding this issue [16]. Population-based studies have methodological limitations that prevent the identification of false positive effects related to this issue because both the lead concentration and symptom severity of ADHD, that are the primary tested variables, are already higher in children with ADHD than in ones without ADHD. To prevent the identification of false positive effects, comparisons of blood lead concentrations with symptom severities should be conducted among medically diagnosed ADHD cases. However, few studies have examined this issue in children with ADHD [17, 18]. Thus far the effect of lead on the symptom severity of ADHD is inconclusive.

Therefore, this hospital-based case-control study was performed to assess the relationship between blood lead concentrations and ADHD. We defined ADHD cases using a semi-structured interview based on the diagnostic criteria of the Diagnostic and Statistical Manual of Mental Disorders-Fourth Edition (DSM-IV) [1]. We also examined the association between blood lead concentrations and symptom severity in ADHD cases. ADHD symptoms were measured with both a parent-reported scale and a computerized cognitive test.

This study has two hypotheses. The primary hypothesis is that children with higher blood lead concentrations will be more likely to be diagnosed with ADHD. The secondary hypothesis is that blood lead concentrations will be associated with the symptom severity of ADHD.

## Methods

### Participants

The present study was designed to frequency-match cases and controls in terms of age and sex. The study was conducted in four university hospitals (Dong-A, Inje, Pusan, and Kosin University) in Busan, Korea, from April to September 2013. All of the participants in this study were recruited in Busan.

The ADHD cases met the following inclusion criteria: (1) diagnosis of ADHD by a child psychiatrist based on the DSM-IV [1]; (2) children between the ages of 6 and 12 years; (3) living in Busan from birth; and (4) provision of written informed consent. Children were excluded from the study based on the following criteria: (1) known history of developmental or neurologic illness, such as mental retardation, cerebral palsy, epilepsy, or pervasive developmental disorder; (2) chronic physical illness, such as a congenital abnormality, endocrine disease, or hematologic disease; (3) previous history of any type of head injury; and (4) inability to provide consent. Non-ADHD controls satisfied the same criteria for enrollment with the exception of the diagnosis of ADHD.

A total of 114 ADHD cases were recruited from child psychiatric clinics at two university hospitals (Donga-A and Inje University). A total of 202 age- and sex-matched controls were recruited from pediatric clinics at three university hospitals (Dong-A, Pusan, and Kosin University). Controls were selected from children with normal physical function, confirmed by pediatrician. After performing the frequency matching by age and sex, a total of 228 children (114 children with ADHD and 114 healthy controls) were ultimately selected.

### Data collection

After the children and their legal guardian provided written informed consent to participate, we acquired information about demographic factors and the children’s medical and developmental histories from the self-administered questionnaires. The variables collected by questionnaires were age (year), sex, gestational age (weeks), birth weight (kilogram), parental education (≤12 years, ≥13 years), parents' smoking behavior (current and/or past smoking, never), and economic status (monthly family income; < $3000, $3000–4000, > $4000).

All children completed the computerized Continuous Performance Test (CPT), and their parents completed the ADHD Rating Scale-IV (ADHD-RS). Next, we conducted diagnostic interviews with each child and parent to confirm the diagnoses of ADHD. Subsequently, we collected blood samples from the children to analyze the blood lead concentrations. Ethical approval for this study was obtained from the Institutional Review Board of the National Cancer Center.

### Diagnosis of ADHD

The diagnoses of ADHD were confirmed based on a semi-structured interview. In the present study, the screening section and the ADHD supplement section of the Korean version of the Kiddie-Schedule for Affective Disorders and Schizophrenia Present and Lifetime Version (K-SADS-PL-K) were used to confirm the diagnoses of ADHD. The K-SADS-PL-K has good to excellent reliability and validity in the diagnosis of ADHD in children [19].

### ADHD Rating Scale-IV

The ADHD-RS is composed of 18 items and scored by a parent [20]. This scale was previously translated into Korean and validated for evaluating symptom severity in Korean children with ADHD according to the DSM-IV diagnostic criteria [21, 22]. This ADHD rating scale uses a four-point response scale that ranges from 0 to 3, and the test items include 9 items that reflect symptoms related to inattentiveness and 9 items that reflect symptoms related to hyperactivity/impulsivity. Higher scores indicate worse attention and impulse inhibition abilities.

### Continuous performance test

Attention and inhibitory control were evaluated with the CPT, which was developed to assess attention and response inhibition in children over 5 year of age [23]. Three sessions with target to non-target ratios of 22, 50 and 78 % were presented, and during each sessions, the subjects responded to or refrained from responding to the target and non-target geometric designs (circle, square, and triangle). Four major variables were assessed: the omission error score, which indicates the number of times that the subject has failed to respond to the target, with high scores reflecting inattention; the commission error score, which indicates the number of times that the subject has produced an incorrect response to the non-target, with high scores reflecting impulsivity; the response time score, which indicates measured time between the presentation of the target stimulus and a correct response, with higher scores reflecting faster in processing speed; and the standard deviation of the response time, which reflects the variability or consistency of attention, with higher scores indicating worse attention and impulse inhibition abilities.

### Blood lead measurement

To determine the levels of lead in the blood, blood samples were obtained from each child via venipuncture in the arm. The blood was drawn into EDTA-containing commercial evacuated tubes (Vacutainer®), and the collected samples were stored at -20 °C prior to analysis. Fifty-microliters aliquots of the blood samples were diluted with 950 μℓ of pretreatment buffer (containing Triton X-100 and ammonium phosphate). The lead levels were measured via graphite furnace atomic absorption spectrometry featuring Zeeman background correction (Varian, USA). Commercial reference materials (Whole Blood Metals Control, Seronorm™, Sero, Norway) were used for internal quality control. The method detection limit of blood lead in the present study was 0.076 μg/dℓ and the instrument detection limit was 0.042 μg/dℓ. The sample was analyzed in triplicate. The coefficient of variation was 4.62 % and the RSD (relative standard deviation) was 2.0 %. All analyses were carried out by Dong-A University Heavy Metal Exposure Environmental Health Center (Busan, Korea). To qualify for equipment assurance, we participated in the German External Quality Assessment Scheme operated by Friedrich-Alexander University.

### Statistical analysis

For the between-group comparisons of the demographic characteristics, Pearson’s χ^2^ tests were used for the nominal variables, and independent t tests were used for the continuous variables.

To test our primary hypothesis, odds ratios (ORs) and 95 % confidence intervals (CIs) were estimated via multivariate logistic regression models using ADHD diagnosis as the dependent variable and the blood lead concentration as the independent variable. The lead concentrations were log-transformed to achieve a normal distribution. The lead levels were then categorized into lead concentration quartiles (first, Q1: 0.18–1.12 μg/dℓ; second, Q2: 1.13–1.71 μg/dℓ; third, Q3: 1.72–2.30 μg/dℓ; and forth quartile, Q4: 2.30–5.35 μg/dℓ). We treated the blood lead concentration as both a continuous (total concentrations) and categorical (quartile) variable.

To test our secondary hypothesis, we conducted multivariate linear regression analysis using ADHD-RS and CPT scores as the dependent variables and the lead concentrations as the independent variable. The regression coefficients were calculated only for the ADHD cases. The covariates considered in analytic models were sex, age, gestational age, birth weight, economic status, parental education, and parents’ smoking behavior.

Statistical significance was evaluated using a two-sided design with alpha set to 0.05. Missing data were excluded from our analysis. All analyses were conducted using IBM SPSS Statistics (version 18.0, SPSS Inc., Chicago, IL).

## Results

### Participant characteristics

The general characteristics of the study population are presented in Table 1. The majority of our study participants were boys. Male-to-female ratio among ADHD cases and matched controls was about 2.5, which is in accordance with epidemiological study of ADHD [24]. The cases and controls did not differ by sex, age, gestational age, birth weight, and parents’ smoking behavior. The proportion of low economic status (< $3000) was higher among the ADHD cases than that among the controls (*p* = 0.01). Duration of education was shorter for parent of the ADHD case than for that of the control (*p* < 0.001). The overall scores for ADHD-RS and CPT, with the exception of response time, were significantly higher in the ADHD cases than in the controls (Table 1).

**Table 1:** General characteristics of study population

	Cases (*n* = 114)	Controls (*n* = 114)	*p-value*
	*n* (%)	*n* (%)	
Sex, male	83 (72.8)	81 (71.1)	0.77
Economic status			
Low (< $3000)	41 (36.3)	63 (55.8)	0.01
Middle ($3000-$4000)	35 (31.0)	30 (26.5)	
High (> $4000)	37 (32.7)	20 (17.7)	
Paternal education			
≥ 13 years	60 (52.6)	93 (81.6)	<0.001
Maternal education			
≥ 13 years	58 (51.3)	94 (82.5)	<0.001
Paternal smoking behavior	60 (52.6)	59 (51.8)	0.50
Maternal smoking behavior	4 (3.5)	1 (0.9)	0.19
	Mean ± SD	Mean ± SD	
Age (year)	8.79 ± 1.57	8.73 ± 1.65	0.77
Gestational age (week)	39.06 ± 1.89	39.12 ± 2.64	0.85
Birth weight (kg)	3.22 ± 0.59	3.30 ± 0.57	0.28
ADHD-Rating Scale			
Inattention	9.33 ± 5.74	3.81 ± 3.50	0.001
Hyperactivity/impulsivity	6.48 ± 4.80	2.35 ± 2.67	0.001
Total	15.82 ± 9.99	6.16 ± 5.65	0.001
Continuous Performance Test			
Omission errors	57.27 ± 18.49	47.53 ± 6.51	0.001
Commission errors	57.36 ± 16.32	52.17 ± 13.76	0.005
Response time	52.33 ± 12.65	51.19 ± 10.58	0.46
Response time variability	53.68 ± 16.84	47.11 ± 8.64	0.001

### Difference in blood lead concentration between the cases and controls

The geometric means (GMs) of the blood lead concentrations were 1.90 ± 0.86 μg/dℓ (range: 0.37–5.35 μg/dℓ) for the cases and 1.59 ± 0.68 μg/dℓ (range: 0.18–3.41 μg/dℓ) for the controls. The children with ADHD exhibited significantly higher blood lead concentrations than the controls with effect size (Cohen’s D) of 0.40 (*p* = 0.003, Table 2).

**Table 2:** Geometric means of blood lead concentration among ADHD cases and controls

Blood lead concentrations	Range (μg/dℓ)	Mean ± SD	*p-value*	Cohen’s D
Cases	0.37–5.35	1.90 ± 0.86	0.003	0.40
Controls	0.18–3.41	1.59 ± 0.68		

### Association between the blood lead concentration and ADHD diagnosis

Table 3 presents the ORs and 95 % CIs for ADHD by the total (continuous) and quartile (categorical) blood lead concentrations. After adjusting for the covariates, the adjusted OR for the log transformed total concentration of blood lead was 1.60 (95 % CI: 1.04–2.45, *p* < 0.05). The analysis also revealed that the children with a blood lead concentrations above 2.30 μg/dℓ were at a 2.5–fold (95 % CI: 1.09–5.87, *p* < 0.05) greater risk of ADHD. The effect of lead concentrations below 2.30 μg/dℓ was not significant.

**Table 3:** Association between blood lead concentrations and ADHD

Blood lead concentrations (range, μg/dℓ)	Crude OR (95 % CI)	*p-value*	Adjusted OR (95 % CI)^a^	*p-value*
Total concentration (0.18–5.35)	1.69 (1.19–2.41)	0.004	1.60 (1.04–2.45)	0.03
Quartile				
1st (0.18–1.12)	Reference		Reference	
2nd (1.13–1.71)	1.38 (0.65–2.90)	0.40	1.26 (0.56–2.84)	0.39
3rd (1.72–2.29)	1.20 (0.57–2.52)	0.63	1.26 (0.55–2.87)	0.61
4th (2.30–5.35)	2.96 (1.38–6.35)	0.005	2.54 (1.09–5.94)	0.03

*OR* odds ratio, *CI* confidence interval

^a^Adjusted for sex, age, gestational age, birth weight, economic status, parental education, and parents’ smoking behavior

### Association between blood lead concentration and ADHD symptom severity

After adjusting for the covariates, our multivariate regression models indicated that blood lead concentrations were not significantly associated with ADHD-RS or CPT profiles the ADHD cases (Table 4).

**Table 4:** Associations between blood lead concentration and the scores of the ADHD-rating scale and continuous performance test within ADHD cases

Variables	Unadjusted B (SE)	*p-value*	Adjusted B (SE)^a^	*p-value*
ADHD-Rating Scale				
Inattention	0.66 (0.62)	0.30	0.69 (0.75)	0.36
Hyperactivity/impulsivity	0.94 (0.52)	0.07	1.04 (0.60)	0.87
Total	1.60 (1.08)	0.14	1.74 (1.29)	0.18
Continuous Performance Test				
Omission errors	0.86 (2.03)	0.67	0.05 (2.29)	0.99
Commission errors	-0.47 (1.79)	0.79	-0.72 (2.08)	0.72
Response time	2.71 (1.36)	0.04	2.48 (1.60)	0.12
Response time variability	2.18 (1.81)	0.24	2.26 (2.23)	0.31

*B* regression coefficient, *SE* standard error

^a^Adjusted for sex, age, gestational age, birth weight, economic status, parental education, and parents’ smoking behavior

## Discussion

Two key findings emerged from the present study. First, even low blood lead exposure is also a potential risk factor of ADHD in children. Second, blood lead concentrations are not associated with the symptom severity of ADHD.

In our study, increased ORs for ADHD were observed at low blood lead concentrations, particularly in the concentration range of 2.30–5.35 μg/dℓ. This result is in close agreement with that of Kim S. et al. [25], who documented the potential risk of a lead exposure of approximately 2 μg/dℓ or higher in relation to medically diagnosed ADHD in children. Previous studies have shown that even low levels of lead exposure are associated with ADHD [13, 26]. However, in the majority of these previous studies, the definition of ADHD relied on subjective rating scales. When investigating associations between potential risk factors and disease, precise definitions of the cases are essential. We enhanced case-defining ability using reliable semi-structured diagnostic tools. The differences in CPT and ARS scores between the cases and controls provided further reliability to case definition. Thus, our findings extend previous evidence supporting the link between lead exposure and ADHD.

Another main finding of the present study is that blood lead concentrations were not significantly associated with either of the symptom domains of ADHD after adjusting for the confounding factors. These findings contrast with those of Nigg et al. [18], who demonstrated that lead exposure is selectively associated with impulsivity, not with inattention in ADHD cases. We need to consider several factors that could affect this discrepancy. The analysis for the effects of blood lead level on ADHD symptoms was conducted within only 114 ADHD cases. The small sample size of our study might limit the statistical power for detecting a correlation. Another possibility may involve the treatment statuses of the ADHD cases. We did not gather information about ADHD treatment histories. It is possible that symptomatically remitted ADHD cases exhibit lower values of ADHD-RS and CPT scores than expected. Thus, the possibility still remains that the effects of the lead level on the symptom domains of ADHD were overshadowed. Another possibility is that the other covariates affected the symptom severity of ADHD. A variety of factors may affect ADHD symptom severity. Recent studies have reported that parental psychopathology, parental skill, comorbidities, and children’s intellectual functions are contributing factors of ADHD symptoms [27]. Our study did not control these factors. Finally, because we excluded diseases which would potentially exhibit inattention or hyperactivity symptoms, it is possible that correlation of ADHD symptoms with blood lead level is attenuated. However, it can also be seen that our exclusion criteria for differential diagnosis of ADHD reduced the likelihood of false positive correlation.

Our results exhibited the negative association between blood lead concentration and CPT commission error, albeit not statistically significant. Some previous studies reported positive association [17, 28], but others reported negative association between blood lead level and CPT commission error [15, 25]. In our study, it is possible that unknown neuropsychological variable such as IQ could affect the CPT performance. Hong et al. demonstrated positive association between blood lead level and impulsivity measured by CTP commission error [28]. However, they also reported the loss of significance of this association after adjusting for IQ. So far, the findings have been inconclusive on selective association between blood lead concentration and impulsivity. Perhaps future studies that measure broad range of neuropsychological function could help clarify the selective association between lead burden and two sets of ADHD symptoms.

The findings of this study must be interpreted within the limitations of the study. First, hospital-based design is subject to selection bias. Our controls recruited from pediatric clinics of three different hospitals. Thus controls in this study would not be representative of the general population. Second, although our study controlled for several covariates, we could not collect the information about intelligence quotients (IQs) and parental history of ADHD. IQ is potential confounder which affects child cognition and behavior. It is possible that IQ would confound ADHD symptoms or affect CPT performance. Although genetic heritability of ADHD is high, it is difficult to assess history of parental ADHD in this study. Diagnosis of ADHD is based on childhood symptoms. We acknowledged that parents could hardly remember inattention or hyperactivity symptoms in their childhood. Furthermore, in our society, ADHD has only in recent decades been recognized by public. Thus, even if they had clinical level of ADHD symptoms in childhood, they rarely have been diagnosed with ADHD. To obtain objective and reliable information of parental ADHD, collateral information from other family members or objective diagnostic tools for adult ADHD are required. Third, we used only a single measure of blood lead level, which limited our ability to investigate the temporal relationship between early life lead exposure and the onset of ADHD. Finally, as noted above, the ADHD treatment histories were omitted from our study.

## Conclusions

This hospital-based case-control study demonstrated that even low blood lead concentration is also a risk factor for ADHD in children. The environmental risk factors for ADHD have meaningful implications for prevention because they are easier to modify than genetic factors. The current consensus is that there is no safe threshold for neurotoxic lead exposure [29]. The findings of our study emphasize the importance of public health policies that reduce the environmental lead exposure. Future studies may be required to ascertain the effects of lead on the symptom severity of ADHD.

## Abbreviations

ADHD: Attention deficit hyperactivity disorder
ADHD-RS: ADHD-rating scale
B: Regression coefficient
CI: Confidence interval
CPT: Continuous performance test
DSM-IV: Diagnostic and Statistical Manual of Mental Disorders-Fourth Edition
GMs: Geometric means
IQ: Intelligence quotient
K-SADS-PL-K: Kiddie-Schedule for Affective Disorders and Schizophrenia-Present and Lifetime Version- Korean version
OR: Odds ration
RSD: Relative standard deviation
SD: Standard deviation
SE: Standard error
